# Observations on the Longevity of the Inhabitants of Ipswich and Hingham, and Proposals for Ascertaining the Value of Estates Held for Life, and the Reversion of Them

**Published:** 1786

**Authors:** Edward Wigglesworth

**Affiliations:** Hollisian Professor of Divinity in the University of Cambridge


XII. *Obſervations on the Longevity of the Inhabitants of Ipſwich and Hingham, and Propoſals for aſcertaining the Value of Eſtates held for Life, and the Reverſion of them. In a Letter from the Rev.* Edward Wiggleſworth, *F. A. A. and Hollifian Profeſſor of Divinity in the Univerſity of Cambridge, to the Honourable* James Bowdoin, *Eſq. Preſ. A. A.* — From the *Memoirs of the American Academy of Arts and Sciences,* Vol. I. 4to. Boſton, 1785.

AFTER the laſt meeting of the Academy, the Rev. Mr. Cutler, of Ipſwich Hamlet, put into my hands a bill of the births and deaths deaths in his pariſh, from September 11, 1771, to September 11, 1781. This bill has been kept with great accuracy; and it ſerves to ſhew, ſo far as a general concluſion can be drawn from the births and deaths in a ſingle pariſh, that either the climate, or the manner of living on the ſea coaſt of New England, is very favourable to life.

From the ſituation of Breſlaw, and the employment of its inhabitants, Dr. Halley ſuppoſes that the deaths in that city are more proper for tracing out the probabilities of the continuance of the human life, in its various ſtages, than thoſe of any other large city in Europe. His table has accordingly been made the ſtandard for eſtimating the value of thoſe eſtates in Great Britain which are held for life.

The Doctor obſerves, that the people of Breſlaw are increaſed by 1238 births annually. Of thoſe it appears, by Dr. Newman's tables, that 348 die yearly, in the firſt year of their age; ſo that about 890 do arrive at a full year's age; and that 193 die in five years, between one and ſix, complete; ſo that 692 of the perſons born ſurvive ſix whole years, and but 710 ſurvive five whole years. Hence it follows, that at Breſlaw five perſons out of twelve that are are born, die before they have completed the fifth year of their age; whereas at Ipswich Hamlet, where, in the course of ten years, 331 persons have been born, but 60 died before they had completed the fifth year, that is but 6 in 33; which determines Ipswich Hamlet to be more than twice as favourable as Breslaw for the preservation of life in its first stages. A similar conclusion may be made with respect to the late periods of life; for, by Dr. Halley's table, out of 1000 persons who die annually at Breslaw, but 34 survive 80 years complete; whereas at Ipswich Hamlet, out of 164 persons who have died in ten years, 21 persons have survived 80 years complete. At the former place, 1 in about 30; at the latter, 1 in about 8 arrive at this great age.

Mr. Lincoln, eldest son of the Hon. Major-general Lincoln, has been so kind as to favour me with a copy of the Rev. Mr. Gay's bills of the baptisms, marriages, and burials, in the first parish in Hingham, from 1726 to 1779, inclusive. This aged and venerable gentleman has been exceedingly accurate in keeping those bills. The age of every person who has died in his parish for fifty four years is set down in the order of the deaths. This bill will be very serviceable viceable in computing a table of the probabilities of the continuance of life in New England, poſſibly more ſo than any others that can be obtained.

This bill I have reduced to the reſpective years of the human life, and by this means have determined the particular number that have died in each age. From the reduction it appears, that Hingham, as well as Ipſwich, is more favourable to longevity than Breſlaw, the Britiſh ſtandard of life. At the firſt pariſh in Hingham, in a period of fifty-four years, 2247 perſons have been born; there have been 521 marriages, and 1113 deaths. Of thoſe, 168 have died in the firſt year, and 404 under five years complete. Out of 1113 perſons who have died in fifty-four years in this pariſh, 84 perſons have ſurvived 80 years complete; whereas at Breſlaw only 34 out of 1000 ſurvived that age.

Theſe ſpeculations are not deſigned as a mere amuſement: they are intended for a valuable purpoſe in civil life. The value of thoſe eſtates which are held for life, and the reverſion of them, can only be determined by knowing the probability which there is that their reſpective holders will live for a longer or ſhorter term term of years. Thofe probabilities are different in different periods of life. The prefent value of two eftates held in dower, whofe annual incomes are equal, may be very different: for inftance, a widow of thirty years of age has an equal chance of living, according to Dr. Halley's table, about twenty-eight years; whereas one of fifty years has only an equal chance of living feventeen years. The prefent value, therefore, of the eftates held by them refpectively, as well as the value of their reverfion, though their annual incomes fhould be equal, are very different. There has as yet been no certain rule eftablifhed for eftimating the value of fuch eftates. Whenever a widow has compounded with the heirs of an eftate for a fum of money in lieu of her dower, the compofition has been made at random, and not on any fixed principles that have determined it to be equitable.

From the comparifon made above, it is evident that the prefent value of eftates among us, held for life, and the value of the reverfion of fuch eftates, cannot be traced with accuracy from the European tables. If ever it fhould be effected, tables muft be conftructed among ourfelves; and this can only be done by keeping regular regular bills of mortality, comparing them together, and making proper deductions from their joint reſult.

*Cambridge,*

January 28, 1782.

